# A Rare Case of Stiff Person Syndrome With Pulmonary Complications

**DOI:** 10.7759/cureus.32631

**Published:** 2022-12-17

**Authors:** Preksha Singh, Shreyans Singhvi, André Crestani, Javier Perez

**Affiliations:** 1 Internal Medicine, Smt. NHL (Nathiba Hargovandas Lakhmichand) Municipal Medical College, Ahmedabad, IND; 2 Internal Medicine, Manidhari Hospital, Jodhpur, IND; 3 Internal Medicine, Pontifical Catholic University of Paraná, Curitiba, BRA; 4 Internal Medicine, Hialeah Hospital, Hialeah, USA

**Keywords:** rare case report, tracheostomy dependence, tracheostomy decannulation (td), ventilator-associated pneumonia, pulmonary complications, rare syndrome, stiff person syndrome

## Abstract

Stiff person syndrome (SPS) is a specific neurological condition, as it’s both rare and unique. SPS is distinguished by muscle rigidity that occurs in waves with simultaneous painful and debilitating muscular spasms. Tactile or auditory stimuli can induce spasms. On electromyographic study, the patient has continuous motor activity, very similar to tetanus. The syndrome can lead to difficulty doing essential daily tasks or even painful conditions like fractures. Apart from clinical signs, some patients have positive anti-glutamic acid decarboxylase (anti-GAD) antibodies, which can also be an excellent confirmatory test for diagnosing SPS. In this case report, we present a 36-year-old female with a long history of SPS, with positive anti-GAD antibodies, leading to her chronic dependence on a tracheostomy tube and the pulmonary complications that followed. The patient suffered from acute encephalopathy secondary to acute respiratory failure. She was placed on a mechanical ventilator due to her respiratory failure but later developed a case of ventilator-associated pneumonia. Respiratory complications have not been reported vividly with this syndrome, so this case sheds light on the same.

## Introduction

Stiff person syndrome (SPS) is an extremely rare and unique disease, with very few cases reported so far. The first case of this syndrome was described in 1956 by Moersch and Woltman at the Mayo Clinic [1,2]. Later, the term for the syndrome was coined as stiff person syndrome by Blum and Jankovic [3]. One of the closest diseases related to this syndrome is tetanus, as in both conditions, there is a central mechanism that affects the peripheral nervous system. Also, both conditions inhibit central gamma-aminobutyric acid (GABA) [4]. GABA is a primary inhibitory neurotransmitter of the central nervous system, and increased neuroexcitation could be a key etiology behind SPS, as seen in tetanus. Clinically, the patient with SPS is distinguished by muscle stiffness that occurs in waves with simultaneous spasms [5,6]. It is frequently observed that rigidity and spasms begin in the axial muscles, usually the neck, back, and shoulders, and later spread to the proximal limb muscles [7]. Although the patient suffering from SPS initially complains of difficulty in movement, later in life, it progresses to multiple problems, which include difficulty in breathing and swallowing, and can even lead to fractures. In our report, we present a woman with a history of SPS for 10 years leading to pulmonary complications.

## Case presentation

A 36-year-old female presented to the emergency room on May 10, 2022. She had been diagnosed with SPS for the last 10 years or so with a positive titer of anti-glutamic acid decarboxylase (anti-GAD) antibodies. She had a negative history of diabetes mellitus. She lived at home with her family and was able to ambulate with assistance, as the patient had an unsteady gait due to her long history of SPS. She required home health assistance for her essential everyday work.

The patient was dependent on tracheotomy and was sent to the emergency room by her pulmonologist for tracheostomy evaluation and examination for decannulation, if possible. The patient also had a percutaneous endoscopic gastronomy (PEG) feeding tube, but it was removed on June 2022, as the patient started to tolerate her meals orally. At the time of admission, she was taking the following medicines for her SPS: baclofen (Lioresal) 20 mg (three times a day), diazepam (Valium) 5 mg/5 ml solution (10 mg every eight hours), Hizentra 4 gm/20 ml (20%) (every 30 days), and immunoglobulin 10% infusion (once a month).

After admission to the ER, the patient was put on a Passy Muir valve (PMV) but was unable to tolerate it. Further, the patient was capped on nasal oxygen, but she deteriorated rapidly, started developing mental changes, and became lethargic with poor arterial blood gas values. Upon this, she was uncapped, transferred to the intensive care unit (ICU) for further management, and placed on a respirator via her tracheostomy. Her chest X-ray and CT scans were normal.

The patient suffered from altered mental status and acute encephalopathy secondary to acute respiratory failure. She suffered respiratory acidosis, unable to tolerate capping or PMV. A CT scan of the brain without IV contrast was performed, where a hyperattenuation of the basilar tip was found, but on further evaluation, it was found to be normal.

The patient was placed on a mechanical ventilator with the following settings: pressure-regulated volume control (PRVC) volume target = 400, rate = 24, positive end-expiratory pressure (PEEP) = 5, and the fraction of inspired oxygen = 50%. The following day, a trial of spontaneous breathing was attempted but after 30 minutes, the patient manifested a pseudo-seizure. The patient tolerated breathing trials better with synchronized intermittent mandatory ventilation (SIMV) than with continuous positive airway pressure (CPAP). The patient continuously remained at risk for life-threatening decompensation. The patient was on diazepam and baclofen previously for her SPS, but her dosage was reduced after being on a ventilator, as she was having respiratory pauses.

Table 1 presents the list of medications the patient was placed on after admission to the ICU.

**Table 1:** List of medication of the patient after admission to the ICU

	Medicine name	Dose	Interval
1	Baclofen (Lioresal) tablet	10 mg	Three times a day
2	Chlorhexidine gluconate (CHG)	Solution	Once a day
3	Diazepam (Valium)	5 mg	8 hourly
4	Mirtazapine (Remeron)	7.5 mg	Once a day
5	Midodrine (Protamine)	5 mg	Thrice a day
6	Pantoprazole (Protonix) delayed-release tablet	40 mg	Daily in the morning
7	Sodium chloride flush 0.9%	10-30 ml flush	8 hourly
8	D5W 1/2 NS (5% dextrose and half normal saline) with potassium chloride	20 mEq/L infusion	20 mL/hr, IV continuous
9	Calcium carbonate table	500 mg	Once a day
10	Calcium chloride 1 gm in dextrose 5%	100 mL/50 mL IV	100 mL/50 mL IV
11	Lidocaine (Xylocaine)	Applied locally	Applied locally
12	Magnesium carbonate (Magonate)	1000 mg/5 ml oral solution	Once a day
13	Magnesium oxide tablet	400 mg	Once a day
14	Metoprolol (Lopressor) injection	2.5 mg	4 hourly
15	Ondansetron hydrochloride (Zofran) injection	4 mg	6 hourly
16	Potassium chloride (Kay Ciel) packet	20-40 mEq	IV
17	Potassium chloride in water	0.2 meQ/ML - IV 10 mEq	IV
18	Acetaminophen (Tylenol) tablet	650 mg	6 hourly

**Figure 1:**
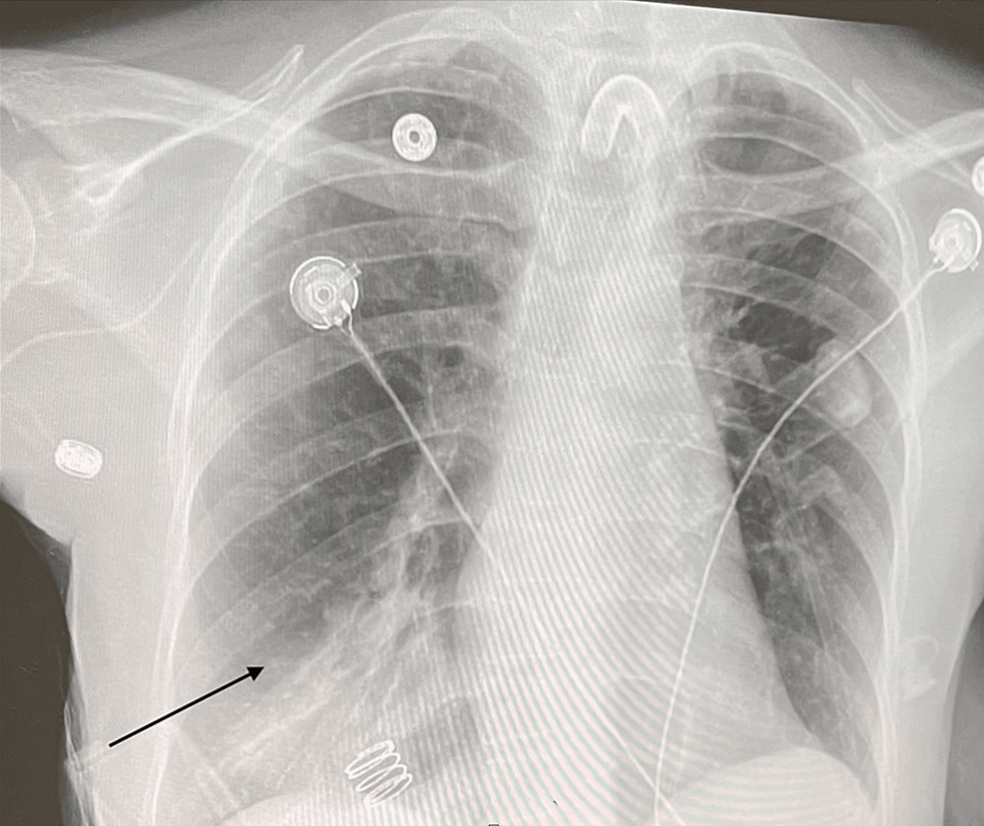
Chest X-ray of the patient presenting right lower lung pneumonia

While the patient was on the ventilator, she developed ventilator-associated right lower lobe pneumonia (Figure 1). The sputum test of the patient was positive for the following pathogens: Staphylococcus aureus (methicillin-sensitive Staphylococcus aureus) and Pseudomonas aeruginosa. The patient was given cefepime and cefazolin for her pneumonia. The patient also developed an episode of hypotension as well as tachycardia. She continued to be monitored on the mechanical ventilator support, with tracheostomy care and hygiene. She was given bronchodilators and a pulmonary toilet. There was continuous monitoring performed for her electrolytes and her renal function. Prophylaxis for deep vein thrombosis was done, and she was being monitored for any further pulmonary complications.

## Discussion

Woltman and Moersch described the first case of SPS in 1956 at the Mayo Clinic. They were also the ones who coined the term "stiff man" syndrome [1,2]. Later, the terminology was changed to "stiff person" syndrome pertaining to the study by Blum and Jankovic [3], as 20 out of 84 cases that they observed were females.

Clinically, the patient with SPS is distinguished by muscle stiffness that occurs in waves with simultaneous spasms [5,6]. As observed in multiple cases, the stiffness and spasms begin in the axial muscles, usually the neck, back, and shoulders, and later spread to the proximal limb muscles [7].

Pathophysiologically, there are multiple etiologies proposed by various case reports; the most commonly proposed is autoimmune, and the basis for the same was raised due to its co-occurrence with other autoimmune disorders, especially diabetes (35% of SPS cases were reported to be diabetic) [7], and other associated autoimmune diseases (vitiligo, celiac sprue, rheumatologic diseases, and thyrogastric disorders) [7,8].

One of the key serum antibodies in this syndrome is anti-GAD antibodies, which were first described in 1988 [9,10]. These anti-GAD antibodies function by inhibiting the activity of the enzyme glutamic acid decarboxylase (GAD), which functions by synthesizing GABA in vitro [2].

GAD is a key enzyme in the synthesis of GABA in the brain, which itself is a primary inhibitory neurotransmitter of the central nervous system. GAD is found in multiple locations in the body, including the central nervous system, pancreatic B-cells, liver, kidney, adrenal glands, ovaries, and testes [11]. Although it is located in multiple locations, primarily, it is located in the central nervous system and the pancreas, which could be a possible explanation for SPS co-occurring with diabetes mellitus.

Although SPS majorly involves muscular rigidity, long-term rigidity can lead to multiple problems, including speech impairment and difficulty swallowing or something as aggressive as painful fractures. In our case, the patient suffered from the consequences of a chronic tracheostomy, which she needed due to her long history of SPS.

There have been multiple treatment options tried for SPS; the most commonly used are baclofen and diazepam, but there have been new reports of immunomodulatory treatment for the syndrome. Azathioprine has been used in some cases of SPS [11,12]. A person with SPS with acute respiratory failure was successfully treated with rituximab [13]. Although new treatment plans are being reported, SPS remains a very debilitating disease for the patient, both physically and mentally.

## Conclusions

In our case, we reported a female patient with SPS and pulmonary complications. Respiratory failure has not been reported vividly with SPS patients, so we believe this case report will be able to provide more information for the same. SPS remains an extremely rare and unique syndrome with very few treatment options and a bad course of the disease. It not only puts the patient under great physical stress but also mental stress. SPS may affect the muscles directly, but it is not just the painful stiffness or spasms the patient suffers from, it is also the disability to perform daily essential work without assistance that can really affect the patient. It continues to progress, as there is only conservative management, and over time, it leads to difficulty in swallowing, breathing, or even painful fractures. It can affect multiple systems. Although there has been some research on the syndrome, there remains a paucity of studies. Thorough research into understanding its etiology would help in forming treatment plans specific to SPS, rather than conservative management.
